# COVID-19 in the endoscopy ward: A potential risk for gastroenterologists

**DOI:** 10.1017/ice.2020.160

**Published:** 2020-04-23

**Authors:** Ahmad Hormati, Mohammad Hadi Karbalaie Niya, Mohammadreza Ghadir, Kamran Bagheri Lankarani, Hossein Ajdarkosh, Fahimeh Safarnezhad Tameshkel, Farhad Zamani

**Affiliations:** 1 Gastroenterology and Hepatology Disease Research Center, Qom University of Medical Sciences, Qom, Iran; 2 Gastrointestinal and Liver Diseases Research Center, Iran University of Medical Sciences, Tehran, Iran; 3 Health Policy Research Center, Shiraz University of Medical Sciences, Fars, Iran

*To the Editor—*COVID-19, an emerging coronavirus disease, is major health problem. As of April 15, 2020, it involved 2,035,299 cases globally, of whom 130,712 died.^1,2^ Coronaviruses comprise a range of positive-sense RNA viruses including several zoonotic viruses. Severe acute respiratory coronavirus type 2 (SARS-CoV-2) has become serious and devastating threat worldwide; it spreads quickly among humans via 2 major routes: respiratory and fecal–oral. Contaminated droplets are the major source of the virus transmission, and the disease initially occurs in the respiratory tract.^3^ Human-to-human transmission by direct contact at <1 m is the most effective way to transfer an infective amount of the virus, and this type of contact occurs frequently in medical centers during routine practice. In addition, the stability of the virus on solid surfaces is high, which puts the medical center setting at high risk for viral contamination.^4^ Thus, medical centers are critical areas for disease control. Unrecognized COVID-19 cases referred for routine endoscopic practice are a probable source of viral contamination of facilities. Endoscopic procedures can facilitate airborne transmission as well as contamination of surfaces. Medical personnel are at risk for disease dissemination by various routes.^5^ Iran, a Middle Eastern country, has a high rate of COVID-19. In Iran, 76,389 confirmed cases and 4,777 deaths have been reported, and Iran ranks first among Middle Eastern countries and eighth in the world for COVID-19 prevalence.^1,2^ Here, we report the COVID-19 prevalence in Iranian endoscopic wards among gastroenterologists.

All gastroenterologists who are on call and who routinely worked in referral hospitals are at risk of viral infection, and unusual presentation of COVID-19 may expose them unexpectedly to the disease.^6,7^ We surveyed these specialists to evaluate the prevalence of COVID-19 and hospitalization using a standard protocol for COVID-19 diagnosis. We distributed a questionnaire to all gastrointestinal wards in Iran, and we received responses from ~480 gastroenterologists by March 26, 2020. Our data analysis revealed that 51 of these gastroenterologists (10.6%) had COVID-19 symptoms that had been confirmed by a laboratory test. Among these cases, 60% had moderate disease, 30% had mild disease, and 10% had severe disease. Furthermore, patients with gastrointestinal symptoms may represent a source of underdiagnosed COVID-19 cases because they do not present with classic symptoms of the disease such as fever and respiratory signs.^6,7^

**Fig. 1.**
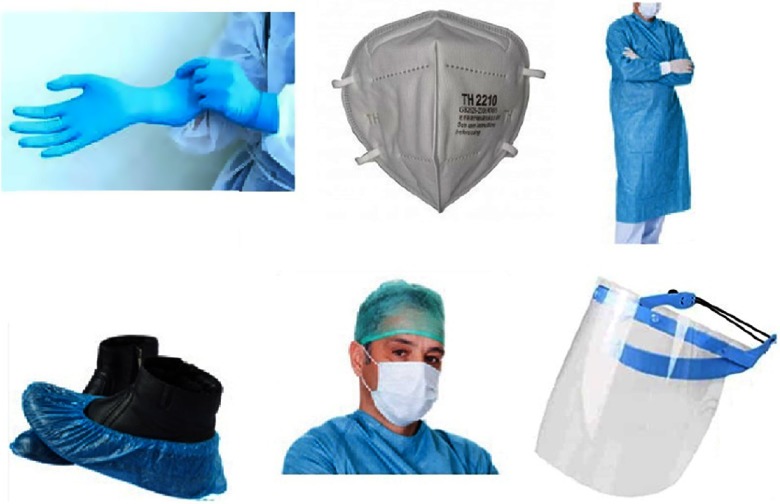
Personal protection equipment (PPE) should used by medical personnel in the endoscopy ward.

Personal protection equipment (PPE) is crucial for preventing exposure to the virus. Various protocols have been recommended for PPE for clinicians as well as patients. Endoscopy patients should wear a special gown, mask, and gloves based on their classification as intermediate- or high-risk patients.^5^ Endoscopy personnel should use standard protective clothing and should maintain a reasonable distance of contact with all patients, especially suspicious cases. The minimal personal protective equipment (PPE) recommended elsewhere should be used, especially by at-risk individuals such as gastroenterologists (Fig. 1).^5^

In conclusion, gastroenterologists, as on-call experts, are at higher risk for COVID-19 via the fecal–oral route as well as the respiratory route during routine practice. Isolation of all patients and strict use of PPE should be observed to reduce the disease burden. In the context of the COVID-19 pandemic, unidentified cases should be detected by implementing more precaution guidelines. For gastrointestinal specialists in endoscopy wards, we highly recommend that they wear at least surgical mask and glasses during clinical visits and that they wear a N95 mask, glasses, a face shield, and latex gloves (not vinyl gloves) during endoscopic procedures.
